# Clinical validation of non-contact vital signs in an emergency department setting

**DOI:** 10.3389/fmedt.2025.1728913

**Published:** 2026-01-20

**Authors:** Amit S. Padaki, Alexa L. Zarzour, Kelly R. Keene, Carlo A. Canepa, Dana R. Levin, Erik L. Antonsen

**Affiliations:** 1 Baylor College of Medicine, Houston, TX, United States; 2 Sam Houston State University College of Osteopathic Medicine, Conroe, TX, United States; 3 Massachusetts General Hospital, Boston, MA, United States

**Keywords:** camera-acquired, emergency department, non-contact, validation, vital signs (MeSH)

## Abstract

**Objectives:**

To evaluate the accuracy of heart rate and respiratory rate acquired by commercially-available device cameras and software.

**Methods:**

One-hundred and eleven subjects were enrolled at an urban academic teaching hospital in Texas. Heart rate (HR) and respiratory rate (RR) measurements were obtained using three commercially-available cameras and processed using software from Presage Technologies. These values were compared to manual counts of a three-lead ECG and end-tidal CO2 from a patient monitor.

**Results:**

The cameras were able to capture HR in 83% of the measurements and RR in 94% of the RR measurements. Camera-acquired HR showed an extremely high correlation with R∼0.99 and a root-mean-square error (RMSE) of 1.62. Respiratory rate showed a high correlation with R∼0.91 and an RMSE of 1.71.

**Conclusions:**

Heart rate and respiratory rate can be accurately acquired using commercially-available camera devices and software for signal processing.

## Introduction

Vital signs are critically important to modern emergency medical care. These metrics [heart rate (HR), respiratory rate (RR), blood pressure, temperature, and pulse oximetry] have been shown to predict a patient's morbidity (1), mortality (2), need for ICU-level admission (3), and suitability for discharge (4). As such, vital signs are used at multiple points of emergency department (ED) care, from triage through treatment through disposition.

Unfortunately, the acquisition of vital signs can be a time-intensive process. Time-and-motion studies have shown the measurement and recording of vital signs may take over five minutes per patient and longer with interruptions (5). Increased crowding, lack of available staff, and length-of-stay have all been shown to delay or decrease the frequency of vital sign measurements (6, 7). Some studies have shown a majority of both children and adults may be missing vital signs during their ED stays (8, 9). Other studies have shown repeat vital signs to be taken less frequently than every two hours (10). With increasing boarding and crowding in ED settings, the frequency of vital sign acquisition may be significantly worse than this reported data.

Many emergency departments have sought to improve vital sign acquisition through various quality improvement initiatives. Some researchers have also evaluated alternative means of vital sign acquisition. Telemedical providers, for instance, have evaluated the accuracy of patients’ self-reported vital signs (11). Device studies have also looked at non-contact vital signs via radar (12) and thermal imaging (13). More recently, studies have evaluated the feasibility of acquiring vital signs using cameras (14).

These studies of camera-acquired vital signs have shown promise, though they are limited by small sample sizes (15, 16). Here, we present the clinical validation of camera-acquired vital signs obtained from commercially available products over a larger sample size.

## Methods

Patients were recruited on a volunteer basis at the Ben Taub Hospital emergency department in Houston, TX. Subjects were required to be between 18 and 75 years of age. Subjects were excluded if they required immediate medical care, had facial scarring, or if they self-reported pregnancy.

One hundred and eleven subjects participated in the study. These included hospital employees and friends and family members of patients in the ED. Thirty-eight were male, seventy-three were female, with ages ranging from 18 to 70 years old. Subjects were predominantly Black and Hispanic. The demography of the study population is shown in Table 1.

**Table 1:** Patient demographics.

Demographic information	(Total *n* = 111)
Gender	
Male	38
Female	73
Age (years)	
Mean	38.8
Range	18–70
Height (inches)	
Mean	66.5
Range	61–75
Weight (lbs)	
Mean	165.3
Range	108–350
Race	
White/Caucasian	3
Black/African American	70
Hispanic	24
Asian/Pacific Islander	9
American Indian/Alaskan	5
Skin Tone (Fitzpatrick Scale)	
1	10
2	14
3	18
4	14
5	29
6	25
Heart rate range	54–106
Respiratory rate range	6–26

**Figure 1:**
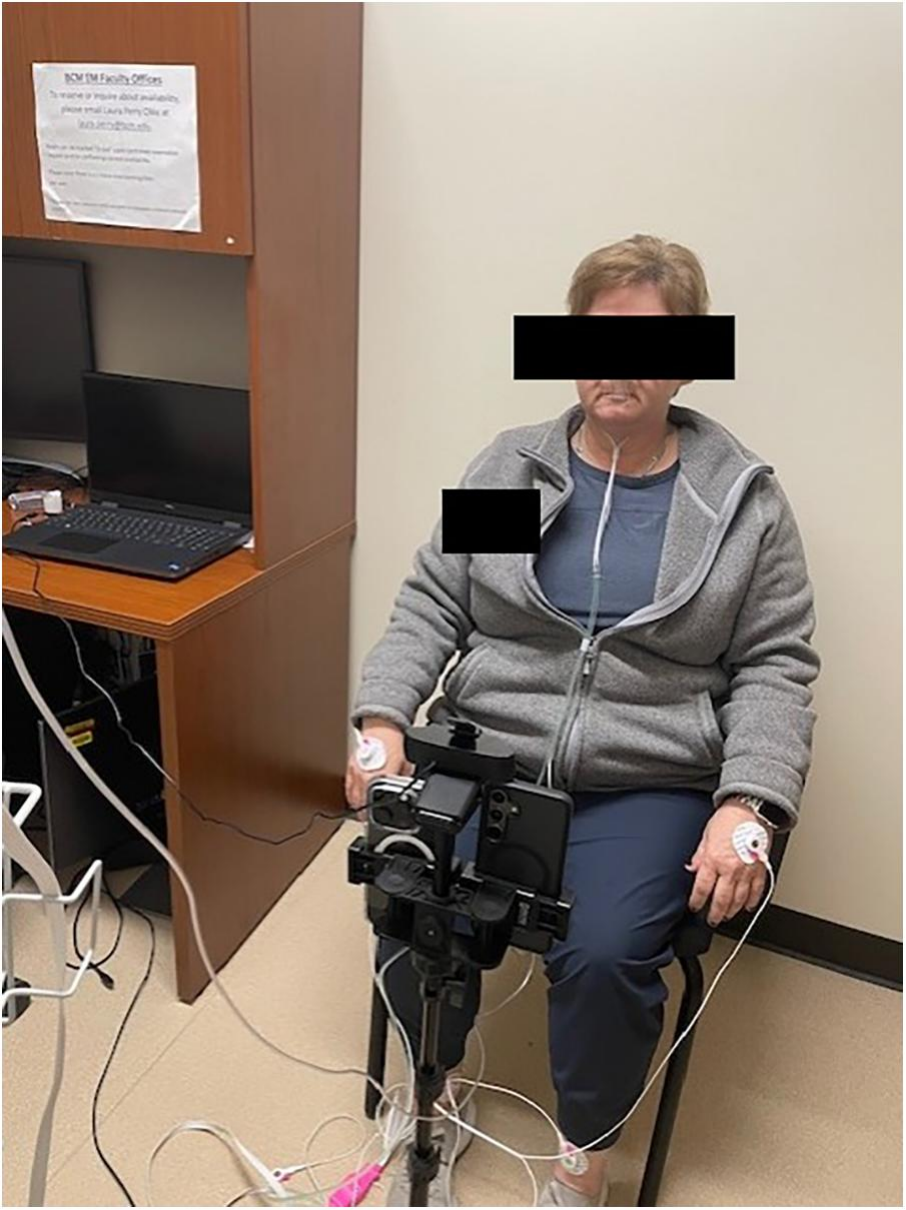
Experimental setup with subject facing three recording cameras.

Subjects were taken to a dedicated room used for the research project. They were seated in a chair and placed on continuous monitoring with a 3-lead ECG and continuous end-tidal capnometry. Subjects were instructed to face the study cameras while keeping their face still within the camera frame. The experimental setup is shown in Figure 1.

Camera data was captured simultaneously with three devices: a Samsung Galaxy S24 Android phone, an iPhone 16 Pro, and a Logitech c920e webcam. The smartphone data was captured over thirty seconds. The webcam data was captured over sixty seconds (thirty seconds overlapping with the smartphones followed by an additional thirty seconds).

The camera data was processed using software from Presage Technologies (Herndon, VA). Signal analysis of pulse plethysmography and chest wall movements were used to calculate heart rate and respiratory rate, respectively. Data processing occurred on the smartphones and a laptop connected to the webcam. Readouts were provided within about thirty seconds. These values are subsequently referred to as Android HR/RR, iPhone HR/RR, and Webcam HR/RR respectively. These measurements were also aggregated into a Pooled HR/RR.

The control data was acquired by a manual count of the waveforms from a Phillips Intellivue MX450 patient monitor over the same time intervals. The presence of a QRS upstroke on the ECG monitor was considered a heartbeat. The downslope of an inhalation on end-tidal capnometry was considered a breath. These values are subsequently referred as control HR/RR. Ten patients were selected at random for review by a second researcher to confirm accuracy of the control measurements.

Statistical analysis was performed using Microsoft Excel and the JASP statistical software. Linear regressions were performed to calculate Pearson correlation coefficients and root-mean-square errors (RMSEs) for the camera-acquired vital signs.

## Results

### Capture rates

278 of 333 possible HR measurements were captured by the software, roughly 83%. 314 of 333 possible RR measurements were captured by the software, roughly 94%. These values were similar across the three devices. The capture rate data is shown in Table 2.

**Table 2:** Camera HR/RR capture rates.

Device	HR capture rate (%)	RR capture rate (%)
Webcam	90/111 (81.1%)	106/111 (95.5%)
Android	96/111 (86.5%)	103/111 (92.8%)
iPhone	92/111 (82.8%)	105/111 (94.6%)
Pooled	278/333 (83.5%)	314/333 (94.3%)

### Correlation and error

Linear regression of the pooled captured HR measurements yielded a correlation coefficient of 0.990 and an RMSE of 1.62. The mean difference was 0.52 beats/minute with an interquartile range (IQR) of 0–1.

Linear regression of the pooled captured RR measurements yielded a correlation coefficient of 0.906 and an RMSE of 1.71. The mean difference was −0.18 breaths/minute with an IQR of −1 to 1.

**Figure 2:**
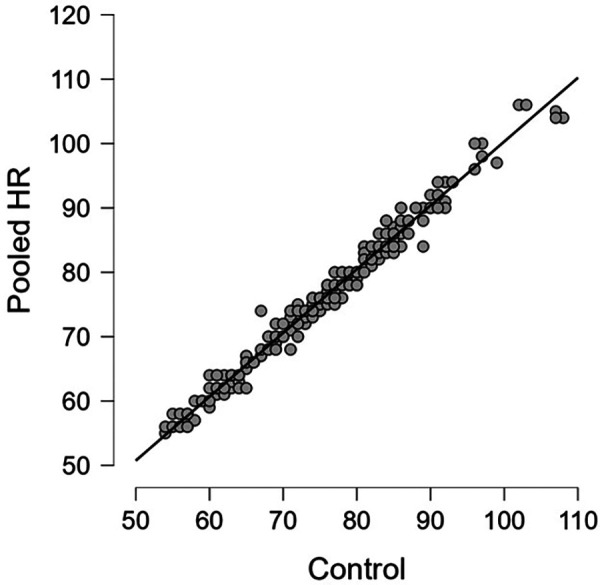
Pooled HR vs. control; R = 0.990.

**Figure 3:**
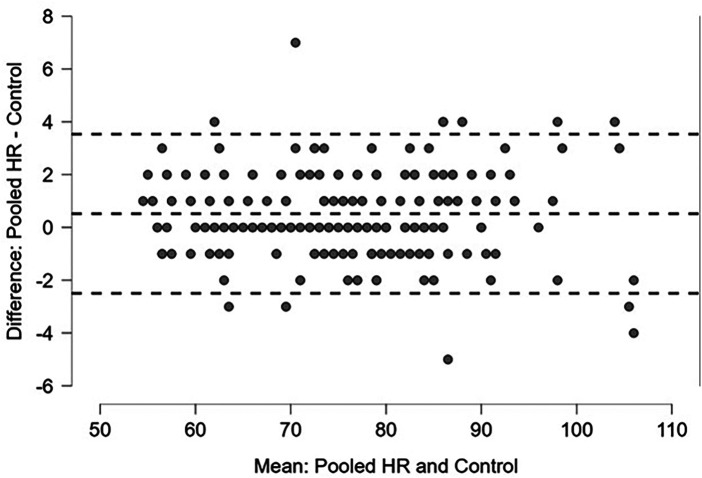
Bland-Altman plot of pooled HR vs. Control [mean difference 0.52, IQR (0, 1)]; dashed lines: +/- 1.96 *σ.*

**Figure 4:**
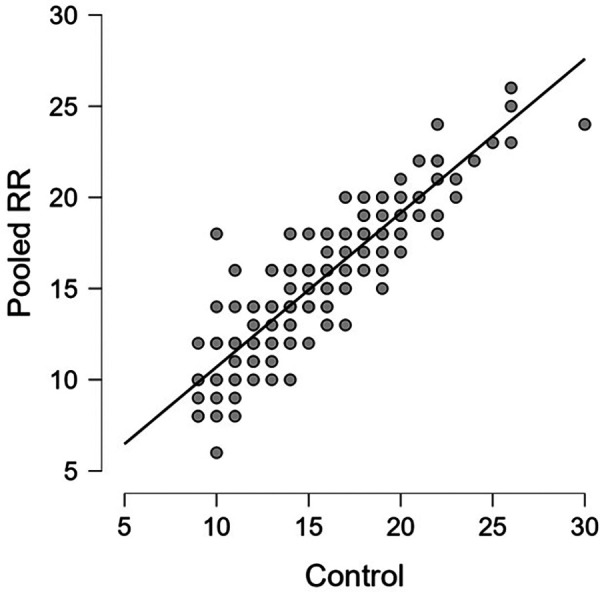
Pooled RR vs. control; R = 0.906.

**Figure 5:**
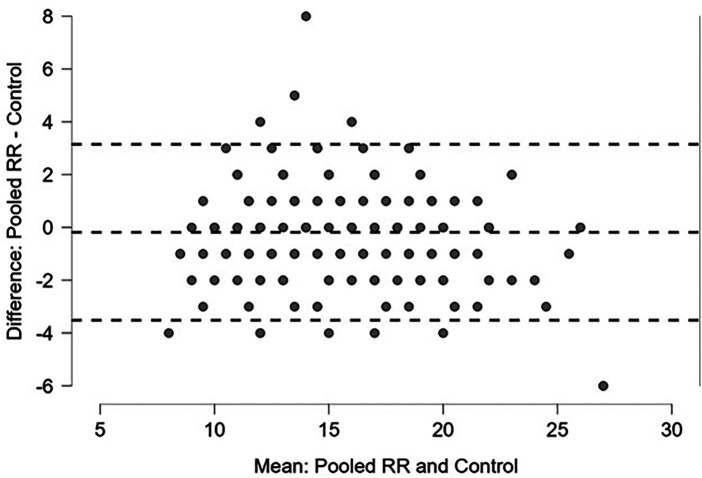
Bland-Altman plot of pooled RR vs. Control [mean −0.18, IQR (−1, 1)]; dashed lines: +/- 1.96 *σ.*

These values are shown in Table 3. Regression and Bland-Altman plots for the HR vs. control are shown in Figures 2, 3. Regression and Bland-Altman plots for the RR vs. control are shown in Figures 4, 5. The random scatter on the Bland-Altman plots for HR and RR suggest that there is good agreement between the methods with no systematic bias or trends.

**Table 3:** Correlation and RMSE of camera-acquired vital signs vs. Control.

Device	HR Correlation	HR RMSE	RR Correlation	RR RMSE
Webcam	.992	1.32	.924	1.75
Android	.991	1.73	.915	1.62
iPhone	.989	1.77	.903	1.74
Pooled	.990	1.62	.906	1.71

### Proportion meeting FDA recommendations

The FDA standard for heart rate measurement in medical devices is accuracy to within 3 beats per minute. 266 of 278 or about 96% of captured measurements would meet this standard. The RMSE of 1.62 would also meet this standard.

The FDA standard for respiratory rate measurement in medical devices is accuracy to within 2 breaths per minute. 280 of 314 or about 89% of captured RR measurements would meet this standard. The RMSE of 1.71 would also meet this standard.

### Interrater reliability

The ten randomly sampled control group measurements were reliably reproduced between the two staff counts. All HR controls were within 1. Nine of ten RR controls were within 1, with the last value being different by 2.

## Discussion

These data show that commercially available cameras can be used to accurately acquire both HR and RR. Camera-acquired HR showed a near-perfect correlation of 0.99. Camera-acquired RR showed a high correlation of 0.91.

These high correlations could improve provider confidence in vital sign accuracy. Respiratory rate, in particular, is known to be measured inconsistently and inaccurately (17). Studies of manual measurement of RR have shown variability in measurement, value bias indicative of estimation, and even outright recording omissions (18). Studies using RR measurements taken from video recordings show only a moderate intraclass correlation coefficients (ICCs) of 0.64–0.662 (19, 20). Latten et al. estimated these inaccurate measurements of RR could then lead to inaccurate scoring in 8%–37% of clinical decision rules (20). The ICC of RR taken from actual patients is likely lower than the ICC of RR taken from video recordings. This would be further complicated by delayed or absent data. The downstream effect on clinical decision making would likely be even more pronounced. Having more consistent and reliable measurements could improve provider confidence in and utilization of RR in emergency department patient management.

This study is subject to several limitations. We first note that this study was performed on generally healthy volunteers. Participants were specifically excluded if they required medical attention. Heart rates ranged from 54 to 106 and respiratory rates ranged from 6 to 26. Additional testing will be needed to evaluate subjects with more abnormal rates. Further, all patients appeared to be in sinus rhythm on the monitor. Additional testing will be needed to evaluate subjects with irregular heart rhythms.

Additionally, this study was performed with fixed cameras and lighting. A prior study of this technology showed lower correlation for both heart rate (R ∼ 0.95) and respiratory rate (R ∼ 0.65) when the technology was used in a less controlled environment (21). (Of note, the prior study used an earlier software version and also used pulse oximetry as a HR control and visual count as an RR control.)

Further, this study provided only HR and RR. Complete vital signs typically include temperature, blood pressure, and pulse oximetry in addition to those provided here.

Future studies could also be expanded in multiple other directions. Subjects could potentially activate the device themselves, allowing for vital sign acquisition without a staff member present. With advances in target recognition and selection, a single mounted camera could be eventually be used to obtain vital sign information regarding multiple subjects in a single room. The available data could be used to trend blood pressure or other physiologic parameters such as compensatory reserve.

Overall, this technology could show benefit in multiple settings. First, it could improve frequency of vital sign acquisition in settings with crowding or low staffing. This could not only improve single-patient care, but also throughput. Second, this technology could improve care in austere and telemedical settings in which no provider would be available to take vital signs at all. Third, this technology could allow for safe vital sign acquisition from a distance in situations where patients or their immediate environments could be unsafe. Improving the software to return parameters beyond heart rate and respiratory rate would increase the potential benefits and use cases further.

## Conclusions

This study shows that heart rate and respiratory can be accurately acquired using commercially available camera devices. Use of this type of non-contact measurement could improve the frequency of vital sign measurements in the ED and clinician trust in vital signs measured in the ED, potentially leading to improved patient care and throughput. This type of study helps lay the foundation for inclusion of camera-based vital signs into clinical workflows in an ER. Additional work is required to document effectiveness in busy clinical settings and the effects on workflow and timing.

## Data Availability

The raw data supporting the conclusions of this article will be made available by the authors, without undue reservation.
